# The Utility of a Digital Virtual Template for Junior Surgeons in Pedicle Screw Placement in the Lumbar Spine

**DOI:** 10.1155/2016/3076025

**Published:** 2016-05-22

**Authors:** Xin Zhao, Jie Zhao, Youzhuan Xie, Jie Mi

**Affiliations:** Department of Orthopaedics, Ninth People's Hospital, Shanghai Jiao Tong University School of Medicine, 639 Zhizaoju Road, Shanghai 200011, China

## Abstract

This study assessed the utility of three-dimensional preoperative image reconstruction as digital virtual templating for junior surgeons in placing a pedicle screw (PS) in the lumbar spine. Twenty-three patients of lumbar disease were operated on with bilateral PS fixation in our hospital. The two sides of lumbar pedicles were randomly divided into “hand-free group” (HFG) and “digital virtual template group” (DVTG) in each patient. Two junior surgeons preoperatively randomly divided into these two groups finished the placement of PSs. The accuracy of PS and the procedure time of PS insertion were recorded. The accuracy of PS in DVTG was 91.8% and that in HFG was 87.7%. The PS insertion procedure time of DVTG was 74.5 ± 8.1 s and that of HFG was 90.9 ± 9.9 s. Although no significant difference was reported in the accurate rate of PS between the two groups, the PS insertion procedure time was significantly shorter in DVTG than in HFG (*P* < 0.05). Digital virtual template is simple and can reduce the procedure time of PS placement.

## 1. Introduction

Because of its anchoring in all three columns, pedicle screws (PSs) are commonly used in rigid fixation in the thoracolumbar spine. The major specific complication of PS placement is that it causes a high risk of bone weakening or lesions of spinal cord, nerve roots, or blood vessels [1, 2]. The majority of cortical violations are found to be clinically silent depending on the location and length of penetration [3, 4]. However, instability of a biomechanical construct and reduced fusion rates may occur due to these initially silent violations [5].

Currently, PS insertion with the hand-free technique under fluoroscopy supervision is a popular method. It is generally safe for experienced surgeons, and its accuracy is high; however, it may be difficult for junior surgeons, and the learning curve is slow [6]. A clinical study reported that the asymptote of the hand-free technique for an inexperienced spinal surgery fellow started after inserting about 80 screws in the learning curve [6].

Three-dimensional (3D) computer-aided navigation can improve the accuracy of PS insertion, especially for minimally invasive transpedicular screw placement [7, 8]. A study showed that a higher cross-sectional percentage fill of the pedicle can be obtained with PS insertion by computer-aided navigation, which is expected to provide greater spinal fixation in instrumented fusion surgery [9]. Although it should be the standard technique of PS insertion in the future, it is costly and occupies a lot of space in the operating room [10]. Finally, only a few hospitals can bear the costs of sensor- or robot-based systems.

Another method for PS insertion is the individual template technique. Various kinds of individual templates for PS placement have been designed. Although it is not as costly as the computer-aided navigation technique, it always needs a long time for preoperative preparation [10, 11].

Digital virtual template is a simple and cost-effective technique [12]; it is extremely popular in China because a picture archiving and communication system (PACS) is generally installed in the department. In the PACS, a surgeon can reconstruct a 3D model for the lumbar in the department. The preoperative digital virtual template in 3D computed tomography (CT) is possible and convenient. It can preoperatively select in an individual the optimal entry point and direction of PS and the correct size of PS. It is beneficial, especially for junior surgeons, to place PSs using a digital virtual template.

In the surgical treatment of severe scoliosis, individual screw placement used entry points preoperatively determined by CT reconstruction and resulted in the improved accuracy and ease of the procedure [13]. However, no literature reports the benefits of using a preoperative digital virtual template for PS insertion by a junior surgeon. The purpose of the study was (1) to assess the use of a digital virtual template in PS insertion in the lumbar by a junior surgeon by measuring the accuracy of the inserted PS and the procedure time and (2) to establish the method of digital virtual template to preoperatively determine entry points.

## 2. Method

From December 2013 to January 2014, 23 consecutive patients of lumbar disease in the spinal surgery department of the hospital were enrolled. The inclusion criteria were as follows: (1) diseases were thoracolumbar fractures, lumbar disk herniation, lumbar canal stenosis, and lumbar spondylolisthesis and (2) patients needed lumbar operation with bilateral PS fixation. The exclusion criteria were as follows: (1) patients had lumbar scoliosis or pedicle deformity and (2) patients had received lumbar operation.

All PSs in the study were inserted by two junior spine surgeons under the direction of an experienced spine surgeon. Each junior surgeon (surgeons <2 years of practice) had experience of less than 20 cases of PS insertion. For medical safety, the experienced spine surgeon (surgeons >10 years of practice) supervised the junior surgeons and ultimately decided whether to reposition a PS. The intraoperative evaluation of all screws includes the PS tract probing and fluoroscopic confirmation. The PS insertion was under fluoroscopy supervision during the operative procedure. The study was approved by the ethics committee of the hospital. All patients signed the informed consent.

In the operation of each patient, the left side and the right side of lumbar pedicles were randomly selected into two groups according to the method of random number table. Lumbar pedicles of one side were referred to as “hand-free group” and those of the other side were referred to as “digital virtual template group.” Two junior surgeons participated in the operations of all patients. In each operation, these two junior surgeons were randomly determined to perform the left or the right side PS fixation.

All patients had preoperative and postoperative CT scans (Philips Brilliance 64 slice CT, Philips Medical Systems, Inc., OH, USA). The preoperative CT scan was used for reconstructing a 3D model of the lumbar in the PACS (GE Healthcare Centricity PACS, General Electricity Company, NY, USA). The ideal entry point and direction of PS can be identified preoperatively with a digital virtual template made on a 3D model. GE Healthcare Centricity Radiology RA 600 software (Centricity Radiology RA 600, General Electricity Company) was used in the PACS, which included digital virtual template software.

### 2.1. Hand-Free Group

In the Weinstein method, the PS is inserted where the entry point is proposed to be at the inferior and lateral corner of the superior articular process. It is identified by using posterior landmarks and in the accessory process. It is removed by Leksell rongeurs. A straight blunt awl is used to find a converging path through the pedicle to the vertebral body. Any sudden advance or persistent resistance indicates that the awl should be repositioned. The hole is palpated by a flexible ball-tip probe. The bony walls and floor of the hole should be intact. After tapping and palpation, the screws are inserted. The anteroposterior and lateral fluoroscopy is used for the position confirmation of the screws.

### 2.2. Digital Virtual Template Group

The entry point is determined preoperatively with 3D CT construction. On the workstation screen, there are four images of the lumbar: a 3D-rotatable dorsal volume rendering (VR) image, a sagittal plane image, a cross-sectional plane image, and a coronal plane image.

**Figure 1:**
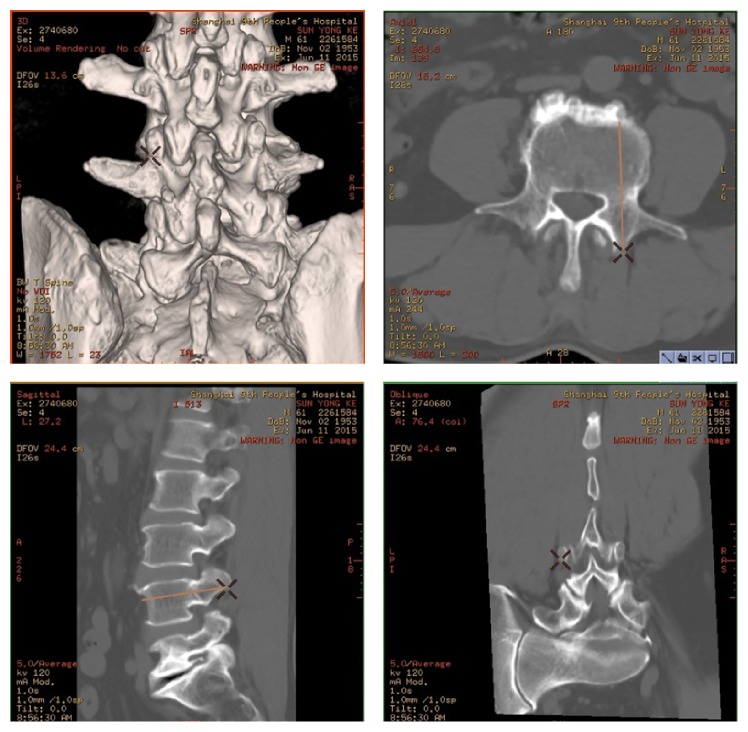
3D CT construction of lumbar spine has four images. By removing the mouse symbol on the surface of the 3D image of the superior articular process, the corresponding points on the other images are removed. When the tangent line of the point divides equally the pedicle on sagittal and cross-sectional plane images simultaneously, the point of mouse indicates an ideal position of the entry point on the surface of the superior articular process.

By removing the mouse symbol on the surface of the 3D image of the superior articular process, the corresponding points on the other images are removed. When the tangent line of the point divides equally the pedicle into sagittal and cross-sectional plane images simultaneously, the point of mouse indicates an ideal position of the entry point on the surface of the superior articular process. Thus, the images containing the position of entry points are recorded by printing on papers and stored in the workstation (Figure 1).

During operation, junior surgeons find the entry point by reading the images. The procedure of the operation is the same as that in the hand-free technique group. Following the natural way of the pedicle, a straight blunt awl is used to find a converging path through the pedicle. The screws are inserted after tapping and palpation. The anteroposterior and lateral fluoroscopy is used for the position confirmation of all the screws.

### 2.3. The Agreement Study

To mark the entry point on the image, a coordinate is established in 3D dorsal VR images. The inferior border of the process is a horizontal coordinate and the medial border of the superior articular process is a longitudinal coordinate. Then, the position of the entry point is localized at the coordinate.

To assess the agreement of the digital virtual template method, the position of the preoperative entry point was templated by an independent spine surgeon and a radiologist repeatedly. The surgeons had 5 years of spine surgery experience, who did not participate in the operation of the study. The distance between the positions of the preoperative entry points templated repeatedly was calculated in the coordinates.

### 2.4. The Breach Rate

The postoperative CT scan was performed 1 week after the operation. The accuracy of PS insertion was evaluated by the postoperative CT scan. All PSs (WeGo Company, Shandong, China) were made of the titanium alloy, which produces minimal artifacts in CT. The breach of PS is defined as follows: Grade 0: no misplacement, or the whole screw is in pedicle; Grade 1: <2 mm and <1/2 diameter of the screw is out of pedicle; Grade 2: >2 and <4 mm or <1 screw diameter is out of pedicle; and Grade 3: >4 mm or whole screw diameter is out of pedicle [14]. Postoperative CT scans were evaluated by the independent spine surgeon and the radiologist referred earlier. They read the postoperative CT together, and a consensus read was established.

The following data were collected during the study: (1) the breach rate, (2) the time of PS insertion, and (3) the distance between the positions of the preoperative entry points templated repeatedly.

### 2.5. Statistical Analysis

All statistical analyses were performed using SPSS. The *P* value less than 0.05 was considered significant. The chi-square test was performed to determine the difference in the breach rates between the two groups. The Student *t*-test (two-tailed, *a* = 0.5) was used to compare the time of PS insertion between the two groups. The one-sample *t*-test was used to compare the distance of the preoperative entry points templated repeatedly. The distance less than 2 mm of the position of the entry points was considered not significant.

## 3. Results

A total of 23 patients (8 females and 15 males) between 35 and 71 years old were enrolled in the study. It included five cases of thoracolumbar factures, four cases of lumbar disk herniation, nine cases of lumbar canal stenosis, and five cases of lumbar spondylolisthesis. A total of 49 PSs were inserted in each group. By the preoperative measurement of the transverse diameters of pedicle on CT, screws of four different diameters were used in the study: 4.5 mm, 5.5 mm, 6.5 mm, and 7.5 mm.

Although the procedure of the placement of PS was under the supervision of the experienced spine surgeon, all PSs in the two groups were inserted successfully without the intervention of the experienced spine surgeon. The evaluation of the postoperative CT revealed that four PSs in the digital template group and six PSs in the HFG group were displaced out of pedicle. The levels of all displaced PSs were of Grade 1 in the two groups. No PS in any groups was of Grade 2 or Grade 3 (Table 1). The accuracy of PS in the digital template group was 91.8% and that in the HFG group was 87.7%. No significant difference (*P* > 0.05) was observed in the two groups (Table 2).

The PS insertion procedure time of the digital group was 74.5 ± 8.1 s and that of the HFG group was 90.9 ± 9.9 s. The procedure time of the digital group was significantly shorter than that of the HFG group (*P* < 0.05) (Table 2).

**Figure 2:**
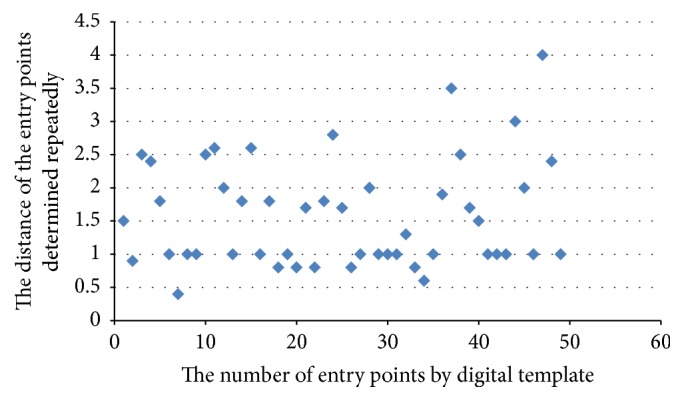
In every pedicle of the digital virtual template group, the distance between the positions of preoperative entry points was determined by different surgeons. The distance less than 2 mm of the position of the entry points was considered not significant.

The distance between the positions of the preoperative entry points determined repeatedly by different doctors was 1.57 ± 0.8 mm. No significant difference was reported in the position of preoperative entry points determined by different doctors (*P* > 0.05) (Figure 2).

## 4. Discussion

This study assessed the utility of a digital virtual template for junior surgeons in PS placement in the lumbar spine. The results showed that the templating technique had good agreement by different doctors. The PS insertion procedure time of the digital group (74.5 ± 8.1 s) was significantly shorter than that of the HFG group (90.9 ± 9.9 s). It reflected that the aid of the virtual digital template could ease the procedure of the insertion of a pedicle screw. Although the accurate rate of PS insertion of the digital group (91.8%) was higher than that of the HFG group (87.7%), no significant difference was reported. A violation of not more than 2 mm was found in any group. According to works of several authors, PS that violates the pedicle cortex by 2 mm or less is considered an acceptably placed screw [15, 16].

With the preoperative 3D construction, junior surgeons can be well familiar with the individual anatomy of the lumbar, especially the posterior surface around the ideal entry point. To know the individual zygapophyseal joints and the position of the ideal entry point well in advance is very crucial for junior surgeons to quickly identify the entry point during the operation procedure. When a surgeon wants to protect the adjacent superior segment facet joint or the paraspinal muscle, the soft tissue dissection around the zygapophyseal joints should be reduced, but then the structure around the ideal entry point cannot be identified with clarity. In this condition, being familiar with the anatomy of the individual preoperatively is important for an inexperienced surgeon.

Besides the aforementioned case, slight anatomic deformities of pedicles are often observed. By any current method to insert PS, the channel through the entry points does not have a large diameter tract. An experienced surgeon can adjust the entry point and the PS direction to insert PS accurately. However, it needs a learning curve for a junior surgeon to master this technique. Some authors think that the asymptote to master the technique for an inexperienced spinal surgery fellow should be started after about 80 screws [6]. With the aid of the digital virtual template, the ideal entry point is identified preoperatively so that the largest security channel is located. This can largely help increase the confidence of junior surgeons, avoid repeated determination of entry points, lower the rupture rate, and reduce the operation time. In this study, the results showed that the PS insertion procedure time of the digital group was significantly shorter than that of the HFG group.

Although the PS insertion procedure time of the digital group was significantly shorter than that of the HFG group, no significant difference was reported in the accurate rate of PS insertion between the two groups. Because of the exclusion criteria, the patients with lumbar scoliosis or pedicle deformity were not enrolled. The lumbar pedicles with complexity anatomy were excluded. Moreover, during the procedure, fluoroscopy was used for position confirmation of all the screws. All these factors guaranteed the high rate of accuracy of PS insertion in the two groups.

Because of the symmetry of the pedicle on both sides, a junior surgeon with the digital virtual template technique can be preoperatively familiar with the individual anatomy of the posterior surface around the ideal entry point of PS on both sides. To reduce study errors, when one junior surgeon finished the PS placement with the digital virtual template technique on one side, the other junior surgeon performed PS insertion with the hand-free technique on the opposite side. These two junior surgeons were randomly selected to place PSs with the digital template technique or the hand-free technique.

In this study, CT images were used to evaluate the accuracy of the position of PSs after operation. X-ray or CT imaging is generally used to assess a PS position; however, CT imaging is currently considered the preferred imaging modality because CT scans have been reported to be more accurate than X-rays [17]. Moreover, the method of CT has high interobserver and intraobserver reliability to evaluate the position of PS after operation [18]. All PSs in this study were made of the titanium alloy, and these PSs were reported to have minimal artifacts in CT than those of stainless steel [19]. The degree of distortion is small and is usually less than 1 mm.

The limitation of the method is how to find the tunnel along the pedicle's direction during the operation. The pedicle's direction is shown on preoperative CT. However, no special tools are available to measure the exact angle during the operation. Moreover, when patients lie prone on the operation table, the position of the actual spine is not exactly the same as that of the virtual spine on the preoperative CT. This leads to the difficulty in accurately following along the pedicle's direction.

This study showed that the aid of a digital virtual template can reduce the procedure time of a junior doctor in placing a PS in the lumbar spine. The PS placement in the lumbar spine is much safer and easier than that in the thoracic and cervical spines. In the lumbar spine, the aid of a digital virtual template may not be necessary for an experienced spine surgeon. Some authors studied the same method in the surgical treatment of severe scoliosis. Most of these scoliosis occurred in the thoracic region. The results showed that PS placement guided by entry points determined by CT reconstruction can result in improved accuracy and ease of the procedure [13].

Computer-aided navigation may be the standard technique of PS insertion in the future. However, it is not popular currently because of its high cost. Obviously, the method of PS placement with the aid of digital template is more simple and convenient. Although it is not the replacement of computer navigation systems in the future, it could largely boost the confidence of junior surgeons to learn the technique of placing a PS in the lumbar spine.

## Figures and Tables

**Table 1:** Pedicle breaches per each level.

	Number of PS		Breach grade of DVTG PS				Breach grade of HFG PS			
	DVTG	HFG	Grade 0	Grade 1	Grade 2	Grade 3	Grade 0	Grade 1	Grade 2	Grade 3
L1	5	5	2	1	0	0	3	0	0	0
L2	5	5	4	1	0	0	4	1	0	0
L3	7	7	6	1	0	0	5	2	0	0
L4	13	13	14	0	0	0	12	1	0	0
L5	14	14	14	1	0	0	14	1	0	0
S1	5	5	5	0	0	0	5	1	0	0
Total	49	49	45	4	0	0	43	6	0	0

PS: pedicle screw; DVTG: digital virtual templating group; HFG: hand-free group.

Grade 0: the whole screw is in pedicle; Grade 1: <2 mm and <1/2 diameter of the screw is out of pedicle; Grade 2: >2 and <4 mm or <1 screw diameter of the screw is out of pedicle; and Grade 3: >4 mm or the whole screw diameter is out of pedicle [14].

**Table 2:** The accuracy and the procedure time of the inserted PS.

	DVTG	HFG
Accuracy rate of PS	91.80%	87.70%
Procedure time of PS insertion	74.5 ± 8.1 s^△^	90.9 ± 9.9 s

^△^Compared with the procedure time of PS insertion in DVTG, *P* < 0.05.

PS: pedicle screw; DVTG: digital virtual template group; HFG: hand-free group.
